# Cardiometabolic risks of SARS-CoV-2 hospitalization using Mendelian Randomization

**DOI:** 10.1038/s41598-021-86757-3

**Published:** 2021-04-12

**Authors:** Noah Lorincz-Comi, Xiaofeng Zhu

**Affiliations:** 1 Department of Population and Quantitative Health Sciences, Case Western Reserve University, Cleveland, OH USA; 2 Department of Population and Quantitative Health Sciences, Case Western Reserve University, Wolstein Research Building Room 1317, 2103 Cornell Rd, Cleveland, OH 44106 USA

**Keywords:** Genetics, Cardiology, Diseases, Medical research, Risk factors

## Abstract

Many cardiometabolic conditions have demonstrated associative evidence with COVID-19 hospitalization risk. However, the observational designs of the studies in which these associations are observed preclude causal inferences of hospitalization risk. Mendelian Randomization (MR) is an alternative risk estimation method more robust to these limitations that allows for causal inferences. We applied four MR methods (MRMix, IMRP, IVW, MREgger) to publicly available GWAS summary statistics from European (COVID-19 GWAS n = 2956) and multi-ethnic populations (COVID-19 GWAS n = 10,908) to better understand extant causal associations between Type II Diabetes (GWAS n = 659,316), BMI (n = 681,275), diastolic and systolic blood pressure, and pulse pressure (n = 757,601 for each) and COVID-19 hospitalization risk across populations. Although no significant causal effect evidence was observed, our data suggested a trend of increasing hospitalization risk for Type II diabetes (IMRP OR, 95% CI 1.67, 0.96–2.92) and pulse pressure (OR, 95% CI 1.27, 0.97–1.66) in the multi-ethnic sample. Type II diabetes and Pulse pressure demonstrates a potential causal association with COVID-19 hospitalization risk, the proper treatment of which may work to reduce the risk of a severe COVID-19 illness requiring hospitalization. However, GWAS of COVID-19 with large sample size is warranted to confirm the causality.

## Introduction

SARS-CoV-2, a novel coronavirus (COVID-19), has globally infected millions of individuals since it first emerged 12 months ago in December 2019^1^. There is tremendous effort to learn more about why some individuals progress from a relatively stable illness to a more serious one requiring hospitalization. Observational studies, while providing some evidence for the conferral of hospitalisation risk by a number of risk factors, have sometimes produced conflicting results^2^. Equally, it would be inappropriate to infer from these studies alone causal associations between a given exposure and COVID-19 hospitalization. The explanations for this vary but may include unresolved bias, confounding, and reverse causation^3,4^. One alternative to observational studies is the randomised controlled trial (RCT) that can effectively eliminate confounding and reverse causation effects via the randomisation process. However, an RCT to identify causal risks for COVID-19 hospitalization would be expensive and unfeasible because it would be impossible to randomize patients based on a hypothesized risk factor in practice. Mendelian randomisation (MR) is an alternative to the RCT that retains the randomisation component but is both feasible and inexpensive^5^. MR capitalizes on the randomisation of an individual’s genetic information during meiosis. This means that the effects of each genetic variant on a given outcome should be less subject to the effects of residual confounding, such as reverse causation since genes are at conception fixed. This makes MR extremely powerful, which could explain its recent popularity in biomedical research.

Observational epidemiological studies have identified Type II Diabetes, hypertension, and body mass index (BMI) as potential risks for COVID-19 hospitalization (vs non-hospitalization). Pooled estimated odds ratios are 2.75 (95% CI 2.09–3.62) for Type II diabetes^6^, 2.30 (1.76–3.00) for hypertension^7^ (binary), and 2.67 (1.52–3.82) for BMI^8^. Not only did all these pooled estimates display heterogeneity in the meta-analysis from which they came, the observational designs of the included studies preclude making any causal inferences. We attempt here to further investigate some of these associations using MR, namely the associations between Type II Diabetes, body mass, diastolic and systolic blood pressure, and pulse pressure. We test for the causal relationships between each of these health conditions and COVID-19 severity, as indicated by hospitalization risk, using publicly available summary statistics from genome-wide association studies (GWAS). These exposures were selected for their hypothesized associations with COVID-19 hospitalization.

## Methods

### Data

COVID-19 summary statistics were retrieved from the Host Genetics Initiative^9^ (covid19hg.rg/results) July 2020 data release for 928 European cases and 2028 controls, and the October 2020 data release for 2430 cases and 8378 controls of European, African, Hispanic, and Middle Eastern ethnicity (12.6% African, 4.7% Hispanic, 6.4% Middle Eastern). Cases were those hospitalised COVID-19 patients and controls were non-hospitalised COVID-19 patients. Alternative control groups were available, but we chose to use non-severe COVID-positive controls as it is the transition from a non-severe to a severe illness that is here of primary interest. Additional analyses using population controls in the COVID-19 GWAS studies are performed, with their results briefly described in the Discussion and more completely presented and described in Supplement Section S1. Since controls were exposed to the SARS-CoV-2, we could reduce the exposure bias. GWAS estimates, from three research groups^10–12^ (UK Biobank, deCODE Genetics, FinnGen), of European individuals were meta-analyzed to yield the complete set of summary statistics for Europeans. GWAS estimates from eight additional research groups^13–20^ (Bonn Study of COVID19 Genetics, SPGRX: Determining the Molecular Pathways and Genetic Predisposition of the Acute Inflammatory Process Caused by SARS-CoV-2, Genes & Health, Penn Medicine Biobank, Qatar Genome Program, Million Veterans Program for Africans, Europeans, and Hispanics, Ancestry, and Biobanque Quebec COVID19; full descriptions of these groups are found at the respective links in the References section) were then analysed with the original three European cohorts to form the expanded set of estimates from more ethnically diverse individuals.

GWAS summary statistics were meta-analysed using an inverse variance-weighting approach to yield a single set of summary statistics for 12,029,423 and 9,503,351 SNPs for European and variable ethnicity populations, respectively. We could not produce a set of summary statistics for strictly non-Europeans as the 20 October 2020 Host Genetics Initiative data release contained GWAS summary statistics for European and non-European individuals that could not be further separated by ethnicity. Only alleles with frequency > 0.01% were included in the European meta-analyses and only those with frequency > 0.1% in the multi-ethnic analyses. Summary statistics for all five exposures, namely diastolic and systolic blood pressure (DBP, SBP), pulse pressure (PP), body mass index (BMI), and Type II diabetes (T2D), were available from public GWAS repositories. All exposure sets included strictly European samples, some of which were from the UK Biobank. It is therefore possible that some individuals contributed genetic information to both the exposure and outcome summary statistic sets. We used PLINK v1.90b6.11^21^ to prune the SNPs and select independent GWAS significant SNPs from the available millions of SNPs for each exposure using the following command: –clump kb 500 –clump-p1 5e−8 –clump-p2 5e−8 –clump-r2 0.1. That is, these SNPs had in their GWAS a p-value less than 5e-8 and a linkage disequilibrium coefficient less than 0.1. For BMI (n = 681,275)^22^, DBP, SBP, PP (n = 757,601 for each)^23^, and Type II Diabetes (n = 659,316)^24^ 1494, 1202, 1134, 926, and 174 SNPs meeting these criteria were identified, respectively. The GWAS data repositories for each exposure are listed in the respective referenced research.

For each exposure, we merged the COVID-19 summary statistics to each exposure data set by SNP. We then harmonised the alleles so that exposure effect alleles corresponded to outcome effect alleles. No SNPs in the original COVID-19 GWAS had between-study heterogenous effect estimates significant at a Bonferroni-adjusted p-value threshold. In the set of summary statistics from only European cohorts, 1186, 947, 884, 723, and 129 selected SNPs (using selection procedures described above) were included in the analyses for BMI, DBP, SBP, PP, and Type II Diabetes, respectively; in cohorts of individuals of variable ethnicity, 1188, 953, 887, 726, and 129 SNPs were selected for BMI, DBP, SBP, PP, and Type II Diabetes, respectively. The European and multi-ethnic COVID GWAS data sets did not contain all the same SNPs. Proxy SNPs were not used to produce the same SNPs in both sets. Lastly, we performed a sensitivity analysis in which we used a linkage disequilibrium coefficient threshold of 0.05 instead of 0.1 in pruning SNPs. The selected SNP counts for these analyses are found in Supplementary Table S3.

### Mendelian randomization analysis

To estimate the causal effects of each exposure on the probability of COVID-19 hospitalization status, we used Mendelian Randomization (MR). This method intends to eliminate potential confounding effects by capitalizing on the random assignment of genetic information at conception. The causal effect of each variant $i$ is generally estimated as ${\widehat{\uptheta }}_{\mathrm{i}}={\widehat{\Gamma }}_{\mathrm{i}}/{\widehat{\upgamma }}_{\mathrm{i}}$ where ${\widehat{\Gamma }}_{i}$ and ${\widehat{\upgamma }}_{\mathrm{i}}$ are the standardized effect estimates of SNP $i$ on the outcome and exposure, respectively. Standardized effect estimates are calculated as

1 $${\widehat{\upgamma }}_{i}={\widehat{\upbeta }}_{E,i}/\left(s{e}_{{\widehat{\upbeta }}_{E,i}}{{n}_{E,i}}^{1/2}\right)$$

for SNP $i$ of the exposure ($E$) and

2 $${\widehat{\Gamma }}_{i}={\widehat{\upbeta }}_{Y,i}{\left[2MA{F}_{i}\left(1-MA{F}_{i}\right)\right]}^{1/2}$$

for the outcome $Y$ where ${MAF}_{i}$ is the minor allele frequency. The standard errors of the effect sizes are also adjusted from the original standard errors in each GWAS as:

3 $${se}_{{\widehat{\upgamma }}_{\mathrm{i}}}={{n}_{E,i}}^{-1/2}, s{e}_{{\widehat{\Gamma }}_{i}}=s{e}_{{\widehat{\upbeta }}_{Y,i}}{\left[2MA{F}_{i}\left(1-MA{F}_{i}\right)\right]}^{1/2}$$

Many methods for estimating the causal effect exist, of which we chose four that are: MRMix^25^, IVW^26^, MR-Egger^27^ and IMRP^28^. Comparing the results of four similar but distinct analytical procedures provided a more complete picture of any existing causal relationships between each exposure and COVID-19 hospitalization. IVW, MR-Egger and IMRP are similar in estimating causal effect but treating horizontal pleiotropic variants differently, which may bias the causal effect estimate if ignored^27^. IVW does not differentiate horizontal pleiotropic variants from vertical pleiotropic variants, MR-Egger is weighted linear regression with an intercept term for adjusting for the horizontal pleiotropic variants, while IMRP identifies horizontal pleiotropic variants and then exclude them through an iterative procedure^28^. MRmix is an estimating equation approach that assumes $\widehat{\Gamma }-\theta \widehat{\gamma }$ follows a normal mixture model, and it does not directly identify horizontal pleiotropic SNPs. IVW is a powerful method but can lead to large bias when horizontal pleiotropic variants are present. MREgger is less susceptible to horizontal pleiotropic variants but can suffer from reduced power. IMRP takes the advantages of both IVW and ME-Egger, and can also account for sample overlap between exposure and outcome data sets, whereas the other methods cannot. MR-PRESSO^29^ also first identifies horizontal pleiotropic variants and estimates causal effect after excluding horizontal pleiotropic variants but uses a computationally intensive simulation approach. Simulation study suggested that IMRP is less biased and computationally more efficient than MR-PRESSO across variety simulation models^28^. MR-TRYX^30^ is a MR approach using multiple traits that are related to exposure and outcome. It adjusts for horizontal pleiotropy by identifying the horizontal pleiotropy pathways but is also more computationally demanding. Although with advantages of using multiple traits, MR-TRYX requires to accurately estimate the effects through pleiotropic pathways, which is a recursive problem. Therefore, MR-PRESSO and MR-TRYX were not used here. All analyses are more fully described elsewhere^31^ and were done using the *MRMix* and *IMRP* packages in R^25,31,32^.

## Results

**Figure 1:**
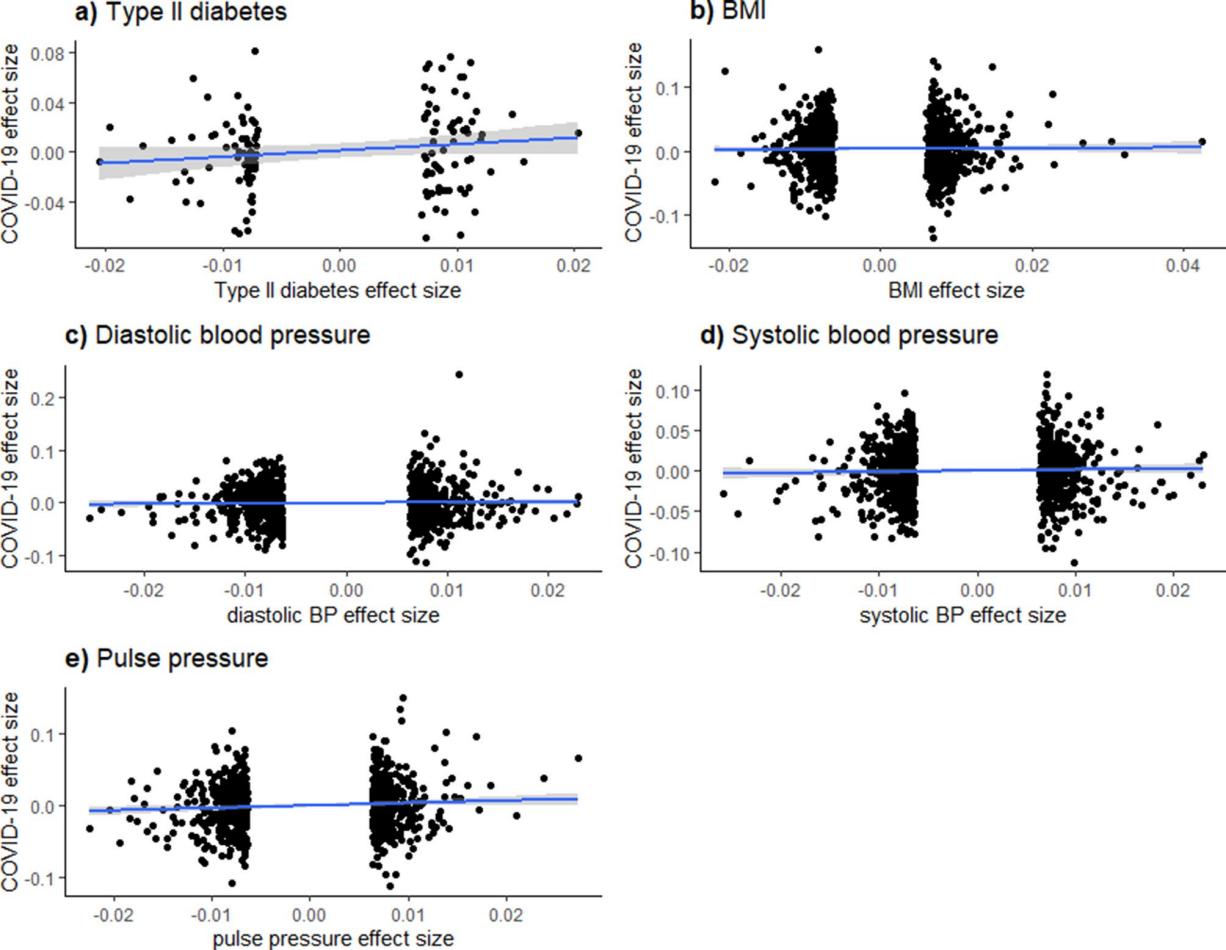
Exposure, COVID-19 Effect size associations—mixed ethnicity sample. These figures display the effect size associations between each exposure (Type II Diabetes, BMI, diastolic and systolic blood pressure, and pulse pressure) and COVID-19 hospitalization risk. Overlaid on the scatterplots are univariate linear regression fitted values and their associated 95% confidence intervals.

Table 1 presents summary statistics for the effect sizes of each exposure and for the correlations between GWAS effect estimate t-statistics for each exposure and COVID-19. These summary statistics indicate that, for each exposure in European only or multiple ethnic sample, the mean exposure effect sizes of only the selected SNPs are all < 0.001. In the European-only sample, the correlations of effect sizes for the respective exposure and COVID-19 hospitalization [i.e., $\widehat{\rho }\left({\widehat{\Gamma }}_{i},{\widehat{\gamma }}_{i}\right)$] are all negative. In the multiple ethnicity sample, the correlations are more robust. These associations are reflected in Fig. 1.

**Table 1:** Summary statistics for sizes of standardized effects of each exposure on COVID-19 hospitalization.

Exposure	Effect size									
	Europeans_a_					Multiple Ethnicity_a_				
	Mean	SD	Min	Max	$\widehat{\rho }$(exposure, COVID-19)_b_ (SE)	Mean	SD	Min	Max	$\widehat{\rho }$(exposure, COVID-19)_b_ (SE)
Type II Diabetes	7.54e−05	0.010	− 0.021	0.020	− 0.033 (0.089)	7.54e−05	0.010	− 0.021	0.020	0.153 (0.084)
BMI	3.99e−04	0.009	− 0.022	0.043	− 0.024 (0.029)	3.79e−04	0.009	− 0.022	0.043	0.013 (0.029)
DBP	− 1.78e−04	0.009	− 0.025	0.023	− 0.035 (0.033)	− 1.59e−04	0.009	− 0.025	0.023	0.026 (0.032)
SBP	− 4.01e−05	0.009	− 0.026	0.023	− 0.027 (0.034)	− 2.63e−05	0.009	− 0.026	0.023	0.036 (0.033)
PP	− 2.47e−04	0.009	− 0.022	0.027	− 0.021 (0.037)	− 2.11e−04	0.009	− 0.022	0.027	0.089 (0.037)

Mean, standard deviation, minimum, and maximum values correspond to the sizes of the standardized effects of the selected SNPs on each exposure, calculated from original GWAS estimates using Eqs. (1) and (3) above. Correlation coefficients represent the correlations between exposure effect sizes and outcome (COVID-19 hospitalization) effect sizes, the latter of which is calculated from the original COVID-19 GWAS using Eqs. (2) and (4) above. All values are presented only for the final selected SNPs (i.e., those with a p-value less than 5e−8 and a linkage disequilibrium coefficient less than 0.1 in the original GWAS). Correlation coefficients (**a**) Estimates from the European set are from 2956 European individuals; estimates from the variable ethnicity set are from 10,908 individuals of either of European, African, Hispanic, or Middle Eastern descent. (**b**) the correlation between the effect sizes for each SNP’s estimated effect on the probability of the respective exposure or COVID-19 hospitalization after all mentioned exclusions (SNP independence, p < 5e−8, Pearson’s r < 0.1 with index SNP) were applied.

**Figure 2:**
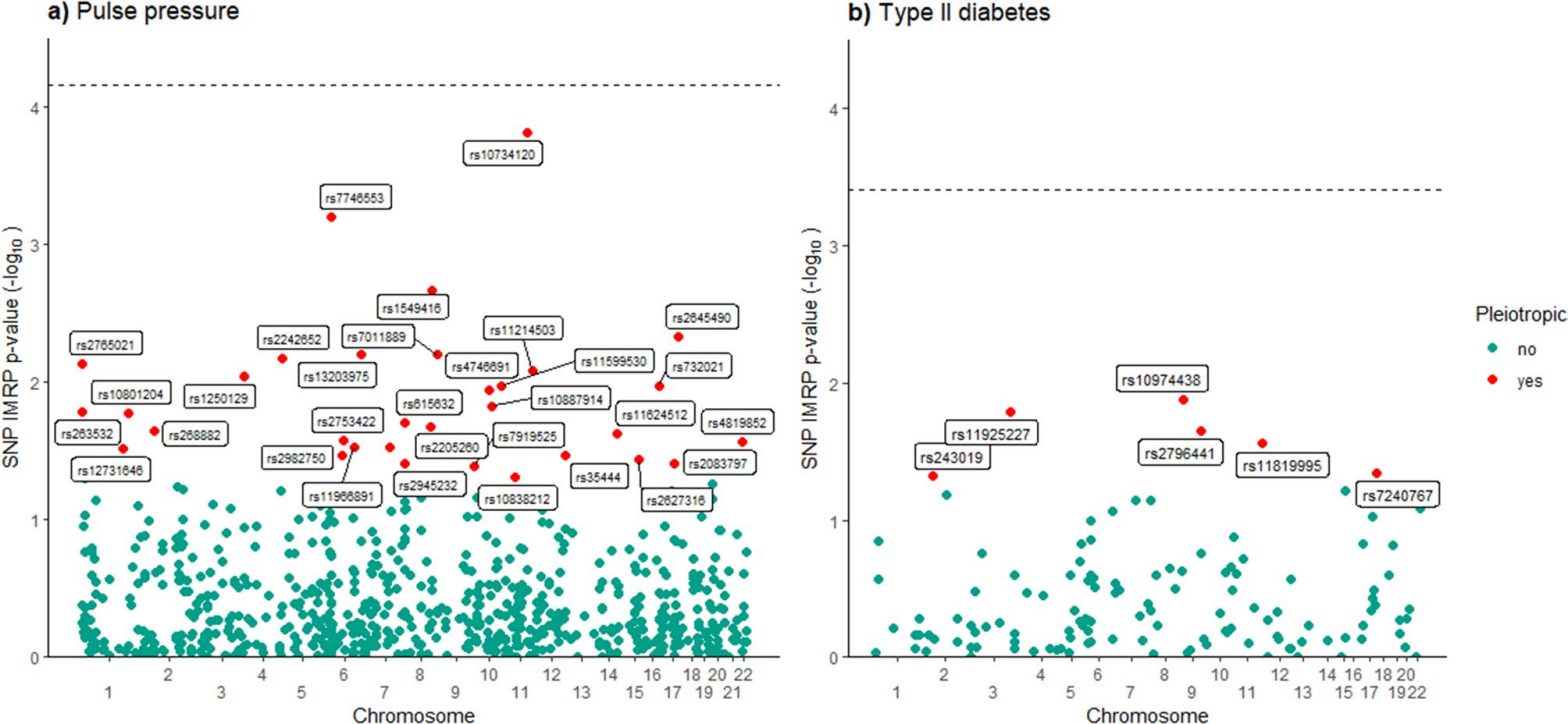
IMRP Pleitropy evidence—mixed ethnicity sample. Displayed are those SNPs demonstrating evidence of pleiotropic effects for pulse pressure and Type II Diabetes in the multi-ethnicity sample as estimated by IMRP. The horizontal dash line represents Bonferroni-adjusted Type I Error rate of p = 0.05.

Using the summary statistics from only European individuals, we did not observe significant causal effects of the exposures to COVID-19 hospitalization after adjusting for multiple tests, although a nominal evidence for BMI was observed in the IMRP analysis (OR = 0.66, 95% CI 0.46–0.96). No uniformly significant causal effect evidence was observed in the multiple ethnicity sample although all the exposures demonstrated increasing risk of COVID-19 hospitalization (Table 2). In the sample of individuals of multiple ethnicities, Type II Diabetes was positively associated with COVID-19 hospitalization using IMRP (1.67, 0.96–2.92). Positive associations are also detected by MRMix, IVW, and MREgger models, though with attenuated effect estimates and wider confidence intervals. Using IMRP, six of the 129 variants in the Type II Diabetes analyses displayed evidence of pleiotropic effects (P < 0.05) which are shown in Fig. 2, although none remain after Bonferroni correction. The intercept term in the MR-Egger model was non-significant (p = 0.784), indicated an absence of pleiotropic effects or low statistical power for detecting them.

**Table 2:** Causal effect estimates of each exposure on COVID-19 hospitalization status.

Exposure/sample	Estimation method			
	MRMix	IMRP	IVW	MREgger
	OR (95% CI)	OR (95% CI)	OR (95% CI)	OR (95% CI)
**European only**				
Type II diabetes	1.00 (1.00, 1.00)	0.55 (0.20, 1.52)	0.85 (0.30, 2.43)	3.15 (0.07, 143.32)
BMI	1.92 (0.03, 129.54)	0.66 (0.46, 0.96)	0.89 (0.62, 1.28)	0.51 (0.15, 1.71)
Diastolic BP	0.78 (0.47, 1.28)	1.04 (0.69, 1.55)	0.81 (0.55, 1.20)	0.75 (0.20, 2.85)
Systolic BP	0.97 (0.60, 1.56)	1.07 (0.70, 1.62)	0.85 (0.56, 1.27)	1.90 (0.47, 7.76)
Pulse pressure	0.92 (0.54, 1.59)	0.86 (0.54, 1.38)	0.88 (0.56, 1.39)	1.85 (0.36, 9.36)
**Mixed ethnicity**				
Type II diabetes	1.11 (1.55e−17, 7.90e16)	1.67 (0.96, 2.92)	1.49 (0.86, 2.56)	1.15 (0.17, 7.91)
BMI	1.32 (0.90, 1.95)	1.08 (0.88, 1.34)	1.12 (0.90, 1.38)	1.74 (0.86, 3.54)
Diastolic BP	0.80 (0.21, 3.02)	1.03 (0.81, 1.30)	1.05 (0.83, 1.32)	1.43 (0.65, 3.15)
Systolic BP	0.67 (8.11e−13, 5.54e11)	1.03 (0.81, 1.31)	1.07 (0.85, 1.36)	1.71 (0.76, 3.87)
Pulse Pressure	1.36 (0.92, 2.02)	1.27 (0.97, 1.66)	1.39 (1.07, 1.81)	3.05 (1.20, 7.77)

Displayed are causal effect estimates for each exposure on COVID-19 hospitalization status within the European sample and multiple ethnicity sample. Estimates are displayed as odds ratios with 95% confidence intervals included in parentheses.

A positive causal association was detected for pulse pressure in the multiple ethnicity sample. The IMRP model produced an estimated odds ratio of 1.27 (95% CI 0.97–1.66). Positive associations are also detected by IVW (1.39, 1.07–1.81) and MR-Egger (3.05, 1.20–7.77), and marginally so for MRMix (1.36, 0.92–2.02). Thirty-two SNPs displayed evidence of pleiotropic effects (P < 0.05) as indicated by IMRP (SNPs included in Fig. 2, detailed in Supplementary Table S1), none of which remained after a Bonferroni adjustment (MR-Egger intercept p-value: 0.088). The results of the sensitivity analysis using a linkage disequilibrium coefficient threshold of 0.05 for pruning SNPs are presented in Supplementary Table S2 and are not meaningfully different from those presented here. All but three of these estimated causal effects are in the same direction as those presented here. Each of these three estimates are nonsignificant in both the main and sensitivity analyses.

**Figure 3:**
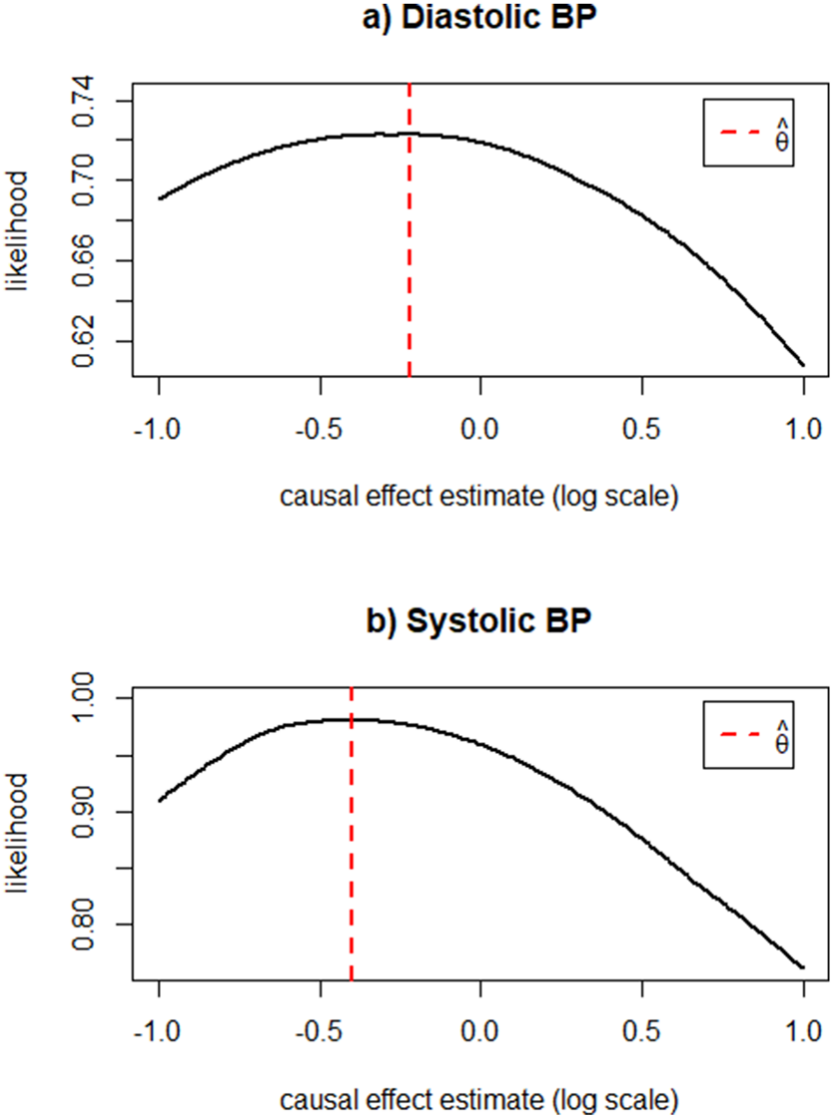
MRMix estimation performance—mixed ethnicity sample. These two plots display the estimated causal effect (theta, $\widehat{\uptheta }$) during maximum likelihood estimation for diastolic and systolic blood pressure by MRMix in the multi-ethnicity sample. A clear, sharp peak indicates stable performance in the estimation of theta. The dotted red lines indicate the ML estimates of theta ($\uptheta$) reported in the Results section, respectively for each exposure.

To better understand why for diastolic and systolic BP in the mixed ethnicity sample MRMix produced a causal effect estimate in the direction opposite of that produced by IMRP, IVW, and MREgger, we also plotted the causal effect estimate ($\widehat{\theta }$) against its likelihood function (see Fig. 3). For both variables a clear but flat peak is present, suggesting large standard error. We also report the MRMix estimation performance for Type II Diabetes and systolic BP in the European sample in Supplementary Figs. S1 and S2 to better understand why the standard errors for these estimates are so large. A possible reason for each of these issues is the small sample size of COVID-19.

## Discussion

Using summary statistics from European-only samples, we failed to detect any compelling evidence supporting any causal associations between hospitalization from COVID-19 and Type II Diabetes, body mass index, diastolic and systolic blood pressure, and pulse pressure. From the multi-ethnic sample, we provide evidence of existing positive causal associations between Type II Diabetes and pulse pressure and the probability of hospitalization from COVID-19. A positive causal association between pulse pressure and COVID-19 hospitalization is biologically plausible as pulse pressure is moderately correlated with lung function (r = − 0.37)^33^, the failure of which is a primary reason for COVID-19 hospitalization. Similarly, although Type II diabetes is associated with a number of risks also hypothesized to be causally associated with COVID-19 severity (e.g., obesity, hypertension, cardiovascular disease), risk for hospitalization may also be conferred because of known inflammatory immune responses in patients with Type II Diabetes^34,35^.

Many other investigations of COVID-19 illness have been completed using Mendelian Randomization methods. For example, Zhang et al.^36^ report no evidence of a causal association between BMI and illness severity (IVW OR, 95% CI, 0.73, 0.12–4.25) whereas Leong et al.^37^ and Ponsford et al.^38^ do (1.66, 1.23–2.25 and 1.47, 1.18–1.83, respectively). Similar to our results, Leong et al. and Ponsford et al. report marginal and no evidence of causal association between systolic blood pressure and illness severity, respectively (1.02, 0.99–1.04 and 0.93, 0.66–1.30) but do report an upward-trending nonsignificant association between Type II Diabetes and illness severity (1.04, 0.95–1.13 and 1.00, 0.91–1.10 [1.09, 0.89–1.33 using MREgger], respectively). Leong et al. also report no evidence of association between diastolic blood pressure and illness severity (0.98, 0.93–1.02). Each of these results are estimated using a range of analysis methods and come from exclusively or overwhelmingly European individuals and population controls. The evidence we present in addition to results from these studies may provide greater rationale for more personalized therapies aimed at reducing hospitalization risk via targeted management of Type II Diabetes and high pulse pressure for individuals with these conditions.

Nonetheless, our study is limited by many factors, not least of which is the relatively small sample sizes from which the COVID-19 hospitalization summary statistics were produced (n = 2956 for only Europeans, 10,908 for variable ethnicities). Results of exposure-specific power analyses indicate that power is low to detect effects for any exposure in the European sample and the multi-ethnicity sample except for Type II Diabetes (43%) and pulse pressure (65%) in the multi-ethnicity sample (see Supplementary Table S4). While we cannot for certain say that this alone can explain some of the null findings, using COVID-19 summary statistics from even larger GWAS samples would have allowed for more accurate estimates of any extant causal associations between each exposure and hospitalization from COVID-19. As more GWAS using COVID-19 patients are completed, more accurate summary statistics produced from larger samples will become publicly available and thus future work will work to reduce the probability of making Type II errors. IVW, MREgger, and MRMix methods may also be subject to residual confounding because of uncontrolled within-person effects arising from sample overlap between the exposure and COVID-19 GWAS.

Additionally, there is the possibility of introduced collider bias because our study controls also have COVID-19^39^. This bias may be introduced through direct or indirect paths from our genetic instruments to COVID-19 incidence. Indirectly, our instruments may be associated with certain non-genetic factors known to confer risk of infection such as BMI, socioeconomic status, male gender, and older age^37,39,40^. Directly, certain allele combinations in SNPs encoding for either *TMPRSS2* or *ACE2* genes may confer additional risk of SARS-CoV-2 susceptibility^41,42^. Using the NCBI dbSNP tool^43^ to identify SNPs in these gene regions, we find that no SNPs in these gene regions were present in any of the final sets of instruments for each exposure and ethnicity sample. This would indicate that our instruments may be free from bias associated with *ACE2* or *TMPRSS2* expression. Alternatively, positive causal associations between BMI and COVID-19 susceptibility have been reported, but associations between Type II Diabetes, systolic and diastolic blood pressure, and pulse pressure have not^39,44^. We further examined whether there were any genetic instrument SNPs overlapped with the genome wide significant variants (p < 5e−8) identified in COVID-19 incidence GWAS using population controls. We examined this using separate GWAS meta-analyses (provided by the Host Genetics Initiative^9^) for European and mixed ethnicity populations. These data and the procedures for this check are reporting in Supplementary Section S1. We did not identify any overlapped SNPs, suggesting the collider bias introduced through the selection of COVID-19 infection is minimal.

Although not statistically significant, the causal effects of risk factors on COVID-19 hospitalization often demonstrated protection effects in Europeans, in contrast with the consistent risk effects in multi-ethnic populations (Table 2). To investigate this discrepancy, we performed MR analysis of risk factors on COVID-19 hospitalization using population controls and using the same sets of instruments (see Supplementary Section S1, Tables S5–S8 for results). In general, all the risk factors demonstrated consistent trends of risk effects on COVID-19 hospitalization in both Europeans or multi-ethnic populations except pulse pressure. Our results suggest that the causal effects of type II diabetes, BMI and blood pressure on COVID-19 hospitalization are likely consistent in Europeans or other ethnic populations when GWAS are obtained by comparing hospitalized COVID-19 patients to the general population. However, COVID-infected Europeans may have a trend of less risk effects than other infected ethnic populations on COVID-19 hospitalization contributed by these risk factors, i.e. type II diabetes, BMI and blood pressure, which may be related to different surveillance of and treatment for COVID-infected individuals.

Our study is strengthened by the very large sizes of the samples from which the exposure summary statistics were drawn. Each of these GWAS included > 650,000 participants. Equally, our study is strengthened by the availability of GWAS summary statistics from different populations of individuals. This of course may allow us to generalize our findings to more people globally. Lastly, the comparison of results across multiple causal effect estimation methods should provide greater confidence in the findings for each exposure. We also benefit from a relatively new method (IMRP) which can detect SNPs displaying evidence of pleiotropic effects. Future work should build on this research to better understand COVID-19 disease burden within and across many populations so as to minimize national and global hospitalization risk.

## Supplementary Information

Supplementary Information.
