# Supplementary material for: Cardiometabolic risks of SARS-CoV-2 hospitalization using Mendelian Randomization
Source: Sci Rep. 2021 Apr 12;11:7848. doi: 10.1038/s41598-021-86757-3 (PMC8042041; doi:10.1038/s41598-021-86757-3)
Supplement: Supplementary file 1 — Supplementary Information. [file 41598_2021_86757_MOESM1_ESM.pdf]

## Cardiometabolic risks of SARS-CoV-2 hospitalization using Mendelian Randomization

Noah Lorincz-Comi, Xiaofeng Zhu

### Supplementary Table S1: Pleiotropy by SNP for Type II Diabetes, Pulse Pressure

| Exposure | SNP | P-value |
|---|---|---|
| ***Type II Diabetes*** | | |
| | rs10974438 | 0.013169 |
| | rs11925227 | 0.016348 |
| | rs2796441 | 0.022327 |
| | rs11819995 | 0.027647 |
| | rs7240767 | 0.044900 |
| | rs243019 | 0.047797 |
| ***Pulse Pressure*** | | |
| | rs10734120 | 0.000152 |
| | rs7746553 | 0.000629 |
| | rs1549416 | 0.002129 |
| | rs2645490 | 0.004694 |
| | rs13203975 | 0.006249 |
| | rs7011889 | 0.006338 |
| | rs2242652 | 0.006652 |
| | rs2765021 | 0.007312 |
| | rs11214503 | 0.008208 |
| | rs1250129 | 0.008968 |
| | rs732021 | 0.010537 |
| | rs11599530 | 0.010602 |
| | rs4746691 | 0.011229 |
| | rs10887914 | 0.014797 |
| | rs263532 | 0.016353 |
| | rs10801204 | 0.016618 |
| | rs615632 | 0.019559 |
| | rs2205260 | 0.021249 |
| | rs268882 | 0.022319 |
| | rs11624512 | 0.023533 |
| | rs2753422 | 0.026566 |
| | rs4819852 | 0.027124 |
| | rs11966891 | 0.029493 |
| | rs7784933 | 0.029511 |
| | rs12731646 | 0.029949 |
| | rs35444 | 0.033905 |
| | rs2982750 | 0.034263 |
| | rs2627316 | 0.036398 |
| | rs2945232 | 0.038756 |
| | rs2083797 | 0.038998 |
| | rs7919525 | 0.040251 |
| | rs10838212 | 0.048706 |

These P-values are from fitted IMRP models using summary statistics from the sample of individuals of multiple ethnicities.

**Supplementary Figure 1**

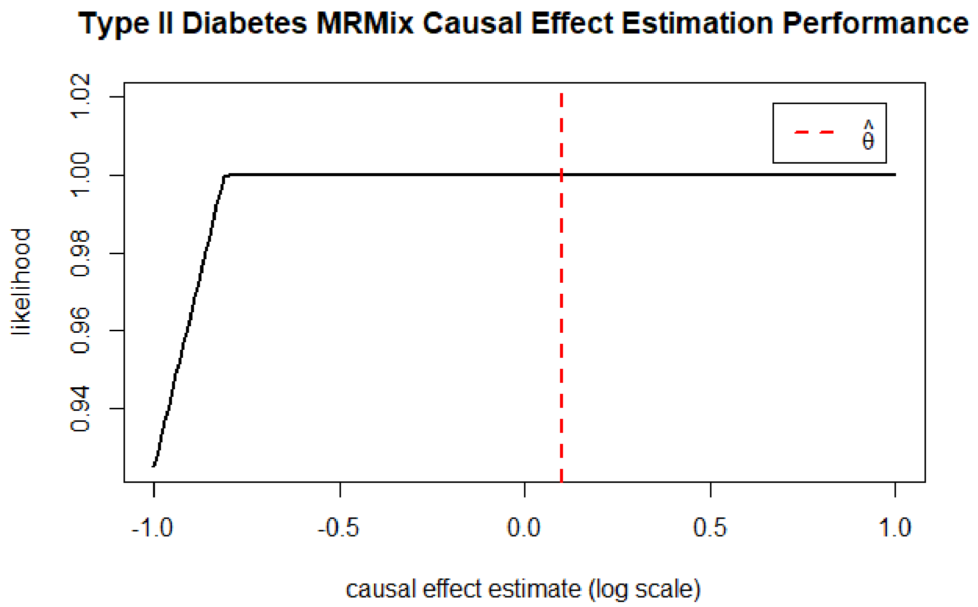


The dashed vertical red line indicates the causal effect estimate produced by MRMix when estimating the causal effect of Type II Diabetes on COVID-19 hospitalization risk. The lack of a unique peak in the likelihood of the causal effect during estimation indicates that the final causal effect estimate is very unstable.

**Supplementary Figure 2**

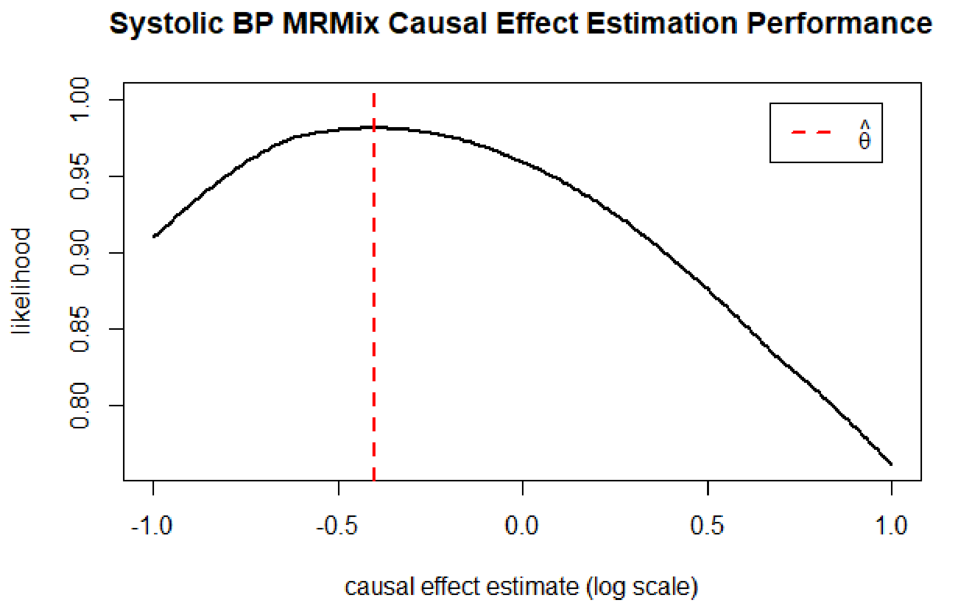

The dashed vertical red line indicates the causal effect estimate produced by MRMix when estimating the causal effect of systolic blood pressure on COVID-19 hospitalization risk. Despite there being a clear peak in the likelihood function during the estimation of the causal effect, the lack of a sharp peak may indicate some issues in the estimation performance of MRMix.

**Supplementary Table S2: Results of sensitivity using linkage disequilibrium coefficient threshold 0.05**

| | Estimation method | | | |
|---|---|---|---|---|
| Exposure/sample | MRMix | IMRP | IVW | MREgger |
| ***European only*** | **OR (95% CI)** | **OR (95% CI)** | **OR (95% CI)** | **OR (95% CI)** |
| Type II Diabetes | 1.00 (0.99, 1.01) | 0.64 (0.22, 1.80) | 1.07 (0.36, 3.21) | 5.26 (0.10, 270.68) |
| BMI | 1.07 (0.28, 4.02) | 0.72 (0.48, 1.07) | 0.97 (0.66, 1.42) | 0.42 (0.12, 1.54) |
| Diastolic BP | 0.79 (0.48, 1.32) | 0.94 (0.61, 1.44) | 0.77 (0.51, 1.18) | 1.06 (0.26, 4.35) |
| Systolic BP | 1.00 (0.99, 1.01) | 1.17 (0.61, 1.44) | 0.93 (0.60, 1.44) | 1.13 (0.26, 4.98) |
| Pulse Pressure | 0.98 (0.35, 2.72) | 0.91 (0.55, 1.51) | 0.89 (0.55, 1.46) | 1.53 (0.28, 8.34) |
| | | | | |
| ***Mixed ethnicity*** | | | | |
| Type II Diabetes | 1.22 (0.03, 50.35) | 1.45 (0.82, 2.59) | 1.29 (0.74, 2.27) | 1.47 (0.21, 10.46) |
| BMI | 1.31 (0.83, 2.06) | 1.06 (0.84, 1.33) | 1.11 (0.89, 1.40) | 1.60 (0.77, 3.33) |
| Diastolic BP | 0.78 (0.27, 2.23) | 1.09 (0.84, 1.40) | 1.13 (0.87, 1.45) | 1.59 (0.68, 3.71) |
| Systolic BP | 0.86 (1.86e-24, 3.95e23) | 1.03 (0.79, 1.34) | 1.08 (0.83, 1.39) | 1.87 (0.79, 4.44) |
| Pulse Pressure | 1.45 (0.72, 2.90) | 1.14 (0.85, 1.53) | 1.31 (0.98, 1.75) | 3.19 (1.18, 8.65) |
| Displayed are causal effect estimates for each exposure on COVID-19 hospitalization status within the European sample and multiple ethnicity sample. Estimates are displayed as odds ratios with 95% confidence intervals included in parentheses. | | | | |

**Supplementary Table S3: Selected SNP counts for main and sensitivity analysis**

| | Linkage disequilibrium coefficient 0.1 | | Linkage disequilibrium coefficient 0.05 | |
|---|---|---|---|---|
| **SNP counts** | **Europeans** | **Mixed ethnicities** | **Europeans** | **Mixed ethnicities** |
| Type II Diabetes | 129 | 129 | 120 | 120 |
| BMI | 1186 | 1188 | 1014 | 1017 |
| Diastolic BP | 947 | 953 | 796 | 801 |
| Systolic BP | 884 | 887 | 740 | 743 |
| Pulse pressure | 723 | 726 | 616 | 619 |
| Selected SNPs are those that had a GWAS p-value less than 5e-8 and a linkage disequilibrium coefficient less than the thresholds indicated in the table: 0.1 and 0.05. | | | | |

**Supplementary Table S4: Power estimation using simulations and MREgger model**

| | Sample | | | |
|---|---|---|---|---|
| | **European only** | | **Mixed ethnicity** | |
| ***Exposure*** | $\hat{\boldsymbol{\beta}}_0$ | $\hat{\boldsymbol{\beta}}_1$ | $\hat{\boldsymbol{\beta}}_0$ | $\hat{\boldsymbol{\beta}}_1$ |
| Type II Diabetes | 0.10 | 0.10 | 0.09 | 0.43 |
| BMI | 0.10 | 0.18 | 0.09 | 0.12 |
| Diastolic BP | 0.09 | 0.22 | 0.10 | 0.17 |
| Systolic BP | 0.09 | 0.17 | 0.08 | 0.22 |
| Pulse Pressure | 0.10 | 0.13 | 0.09 | 0.65 |
| These values represent the estimated power available to observe significant pleiotropic and causal effect estimates. $\hat{\boldsymbol{\beta}}_0$ and $\hat{\boldsymbol{\beta}}_1$ represent the pleiotropic and causal effect estimates, respectively, under an MREgger model. | | | | |

We estimated power using an MREgger model because it allowed us to test for pleiotropic and non-pleiotropic SNP effects efficiently and simultaneously. Available power was estimated using a simulation procedure that followed these steps for each exposure:

1) Set $\mu_{\hat{\gamma}}$, $\mu_{\hat{\Gamma}}$, $\sigma^2_{\hat{\gamma}}$, $\sigma^2_{\hat{\Gamma}}$, $\sigma_{\hat{\gamma}\hat{\Gamma}}$, and $n_{SNPs}$ (the number of selected SNPs included in analyses) to their exposure-specific observed values
    a. Note: $\hat{\gamma}$ and $\hat{\Gamma}$ were normalized [i.e., made to be distributed as approximately $N(0,1)$] before calculating means, variances, and covariances. This removes the requirement to include SNP weights.
2) Draw random sample of size $n_{SNPs}$ from $(\hat{\gamma}, \hat{\Gamma}) \sim N\left(\begin{bmatrix}\mu_{\hat{\gamma}} \\ \mu_{\hat{\Gamma}}\end{bmatrix}, \begin{bmatrix}\sigma^2_{\hat{\gamma}} & \sigma_{\hat{\gamma}\hat{\Gamma}} \\ \sigma_{\hat{\gamma}\hat{\Gamma}} & \sigma^2_{\hat{\Gamma}}\end{bmatrix}\right)$
3) Fit MREgger model $\hat{\Gamma}_i = \hat{\beta}_0 + \hat{\beta}_1 \hat{\gamma}_i + e_i$ and retain test statistics as $t^*_{\hat{\beta}_0, 0.05/2, n-2, b}$ and $t^*_{\hat{\beta}_1, 0.05/2, n-2, b}$ for $\hat{\beta}^*_0$ and $\hat{\beta}^*_1, respectively$
4) Repeat steps 2 – 3 $B = 5000$ times
5) Power for $\hat{\beta}^*_0$, $\hat{\beta}^*_1$ respectively approximately equal to $\#\{t^*_{\hat{\beta}_0, 0.05/2, n-2} < 0.05\}/B$, $\#\{t^*_{\hat{\beta}_1, 0.05/2, n-2} < 0.05\}/B$, or the respective proportion of significant effect estimates across the 5000 replications

**Supplementary Section S1**

We also sought to understand the associations between Type II Diabetes, BMI, diastolic and systolic blood pressure, and pulse pressure and COVID-19 hospitalization using a new GWAS set of summary statistics produced using control subjects from the general population. In our main analyses, COVID-19 GWAS control subjects were COVID-19 positive. The GWAS summary statistics for European and mixed ethnicity samples using population controls for the COVID-19 hospitalization analysis are from the Host Genetics Initiative[1] 18 January 2021 releases named `B2_ALL_eur` (9,986 cases, 1,877,672 controls) and `B2_ALL_leave_23andme` (12,888 cases, 1,295,966 controls; 92.2% European, 1.6% African, 1.3% East Asian, 1.0% Middle Eastern, 3.4% Hispanic, and 0.5% American), respectively. The mixed ethnicity sample has a smaller sample size because the European sample contains data from 23andMe[2] whereas the mixed ethnicity sample does not. There is no available European sample without the 23andMe data. To provide more evidence that our results may not be biased from an uncontrolled collider (i.e., COVID-19 infection), we verified that no SNPs that reached genome-wide significance (i.e., p<5e-8) in either of the COVID-19 GWAS using population controls were present in any of the selected instruments used in our analyses. We additionally report the correlation coefficients between exposure and COVID-19 effect sizes when the COVID-19 GWAS uses control subjects from the general population (see Supplementary Table S5).

**Supplementary Table S5: Correlations between exposure, COVID-19 effect sizes in GWAS using population controls**

| | Control group | | | |
|---|---|---|---|---|
| | COVID-19 positive controls | | Population controls | |
| ***Exposure*** | **European** | **Mixed ethnicity** | **European** | **Mixed Ethnicity** |
| Type II Diabetes | -0.033 (0.089) | 0.153 (0.084) | 0.040 (0.087) | 0.131 (0.085) |
| BMI | -0.024 (0.029) | 0.013 (0.029) | 0.221 (0.027) | 0.280 (0.027) |
| Diastolic BP | -0.035 (0.033) | 0.026 (0.032) | 0.035 (0.032) | 0.006 (0.033) |
| Systolic BP | -0.027 (0.034) | 0.036 (0.033) | 0.047 (0.034) | 0.038 (0.034) |
| Pulse Pressure | -0.021 (0.037) | 0.089 (0.037) | -0.004 (0.037) | -0.007 (0.037) |
| These values represent the Pearson correlation coefficients (and their standard errors in parentheses) between exposure and COVID-19 effect sizes. | | | | |

If collider bias were artificially inflating our observed effect sizes, we may expect that, when using a COVID-19 GWAS sample using population controls, that the effect size correlations would be smaller (since a collider-corrected causal effect estimate would be an attenuated version of the naïve, collider-uncorrected estimate[3]). However, collider bias is not the only factor that may produce differences in correlations between effect sizes under different COVID-19 control subject designs. The estimates for Type II Diabetes, BMI, and pulse pressure (both in European and mixed ethnicity samples) here are different from the corresponding estimates when using COVID-19 positive controls. For BMI, these correlations are higher in both samples when using population controls compared to COVID-19 positive controls. For Type II Diabetes and pulse pressure, these correlations are smaller in absolute value in both samples when using population controls compared to COVID-19

positive controls. Of greater relevance is the opposing directions of association within the European samples and between the control types. That is, when using COVID-19 positive controls, all exposures appear weakly negatively associated with COVID-19 hospitalization. When using population controls in the COVID-19 GWAS, all exposures except pulse pressure appear weakly positively associated with hospitalization from COVID-19. These differences motivated us to complete additional Mendelian Randomization analyses under the same conditions as in our main analyses but now using COVID-19 GWAS that used population controls instead of COVID-19 positive controls.  As before, we report the results from MRMix, IMRP, IVW, and MREgger models. First, we present the degree of sample overlap between each exposure and new COVID-19 GWAS data sets expressed as the correlations between GWAS test statistics for the SNP-level effect estimate (see Supplementary Table S6).

**Supplementary Table S6: Correlations between all exposure, outcome SNPs to detect sample overlap**

| | COVID positive controls | | Population controls | |
|---|---|---|---|---|
| | Sample | | Sample | |
| | **European** | **Mixed ethnicity** | **European** | **Mixed ethnicity** |
| Type II Diabetes | 0.006 | 0.023 | -0.028 | -0.032 |
| BMI | 0.004 | 0.016 | 0.067 | 0.080 |
| Diastolic BP | -0.010 | 0.003 | 0.003 | 0.002 |
| Systolic BP | -0.011 | 0.002 | -0.001 | -0.001 |
| Pulse Pressure | -0.010 | 0.000 | -0.007 | -0.005 |
| These values represent the Pearson correlation coefficients between the GWAS effect estimates from all SNPs present in both the respective exposure and COVID-19 GWAS sets of summary statistics. These correlations are used by IMRP to control for sample overlap. | | | | |

We next harmonized the same selected SNPs used earlier in the main analyses (i.e., when we used the COVID-19 GWAS from COVID-19 positive controls) with the full list of SNPs from the new COVID-19 GWAS using population controls. These selected SNP counts are displayed in Supplementary Table S7. The only explanation for different SNP counts when using COVDI-19 positive controls vs general population controls is that the COVID-19 GWAS for population controls did not initially have all the same SNPs as the COVID-19 GWAS for COVID-19 positive controls.

**Supplementary Table S7: Selected SNP counts for each exposure, sample, using population controls**

| | Sample | |
|---|---|---|
| | **European** | **Mixed ethnicity** |
| Type II Diabetes | 129 | 128 |
| BMI | 1178 | 1171 |
| Diastolic BP | 944 | 931 |
| Systolic BP | 880 | 868 |
| Pulse pressure | 724 | 716 |

These values are the counts of selected SNPs used as instruments in the mendelian randomization analyses using COVID-19 GWAS that used control subjects from the general population. These may be compared to the SNP counts in Supplementary Table S3 for COVID-19 GWAS completed using COVID-19 positive control subjects. There are no meaningful differences in these two sets of SNP counts. These SNPs were selected using a linkage disequilibrium coefficient of $r^2<0.1$ and this PLINK command: --clump kb 500 –clump-p1 5e-8 –clump-p2 5e-8 –clump-r2 0.1

We next fit MRMix, IMRP, IVW, and MREgger models with these selected SNPs harmonised from the COVID-19 GWAS using individuals from the general population as control subjects. These results are displayed in Supplementary Table S8.

**Supplementary Table S8: Results of MR analyses using population controls**

| | Estimation method | | | |
|---|---|---|---|---|
| Exposure/sample | MRMix | IMRP | IVW | MREgger |
| ***European only*** | **OR (95% CI)** | **OR (95% CI)** | **OR (95% CI)** | **OR (95% CI)** |
| Type II Diabetes | 1.078 (<0.01, 6.94e12) | 1.215 (0.952, 1.549) | 1.069 (0.847, 1.349) | 0.497 (0.217, 1.141) |
| BMI | 1.507 (0.989, 2.297) | 1.431 (1.306, 1.569) | 1.449 (1.323, 1.588) | 1.629 (1.200, 2.212) |
| Diastolic BP | 1.116 (1.095, 1.211) | 1.064 (0.990, 1.211) | 1.064 (0.958, 1.182) | 1.121 (0.785, 1.602) |
| Systolic BP | 0.961 (0.698, 1.323) | 1.037 (0.935, 1.150) | 1.072 (0.965, 1.192) | 1.166 (0.812, 1.675) |
| Pulse Pressure | 0.942 (0.720, 1.231) | 0.959 (0.854, 1.076) | 0.996 (0.887, 1.119) | 0.967 (0.643, 1.454) |
| | | | | |
| ***Mixed ethnicity*** | | | | |
| Type II Diabetes | 1.584 (0.020, 123.978) | 1.245 (1.011, 1.534) | 1.163 (0.942, 1.435) | 0.499 (0.238, 1.046) |
| BMI | 1.649 (1.098, 2.476) | 1.532 (1.414, 1.660) | 1.523 (1.405, 1.651) | 1.727 (1.320, 2.259) |
| Diastolic BP | 1.000 (0.830, 1.204) | 1.046 (0.958, 1.143) | 1.015 (0.926, 1.112) | 1.058 (0.776, 1.443) |
| Systolic BP | 1.020 (0.717, 1.452) | 1.035 (0.946, 1.133) | 1.051 (0.958, 1.153) | 1.032 (0.751, 1.417) |
| Pulse Pressure | 0.942 (0.727, 1.219) | 0.969 (0.875, 1.074) | 0.987 (0.887, 1.098) | 0.989 (0.679, 1.441) |

Displayed are causal effect estimates for each exposure on COVID-19 hospitalization status within the European and multiple ethnicity samples where the COVID-19 GWAS effect estimates are produced using population controls. Estimates are displayed as odds ratios with 95% confidence intervals included in parentheses. The European sample is from 1,887,658 individuals and the mixed ethnicity sample is from 1.308,854 individuals.

These analyses help us learn more about the risk factors associated with hospitalization from COVID-19 in the general population. These results provide evidence (of varying degrees) indicating that in both samples Type II Diabetes, BMI, and diastolic and systolic blood pressure are positively causally associated with COVID-19 hospitalization in the general population. Causal effect estimates are generally consistent within and between the European and mixed ethnicity samples for each exposure. These results may be compared to those produced by our main analysis intended to learn more about the risk factors associated with hospitalization from COVID-19 in the population of individuals with SARS-CoV-2 infection. It appears that risk of hospitalization from COVID-19 in the general population is consistently conferred by these exposures in European and mixed ethnicity populations. However, as demonstrated in our main analyses, the exposures conferring risk of hospitalization in the population of SARS-CoV-2 infected individuals appear different in European vs mixed ethnicity populations. That is, in our main analysis using COVID-19 positive controls, the causal effect estimates produced in the European sample are not generally replicated in

the mixed ethnicity sample. Alternatively, in this analysis using controls from the general population, the risks of hospitalization from COVID-19 in the European sample are generally replicated in the mixed ethnicity sample. Using COVID-19 positive controls, exposures in the European sample are all nonsignificant and mixed in their direction of effect whereas exposures in the mixed ethnicity sample are almost all positively associated with hospitalization risk. We may thus conclude that, once infected with SARS-CoV-2, these exposures confer greater risk of hospitalization for non-European populations compared to European populations. We may observe this difference because of general differences in resources (e.g., socioeconomic capital, healthcare access/utilization) between European and non-European populations, although further investigation is required. Alternatively, compared to the general population, the risks of hospitalization conferred by these exposures are consistently positive (except pulse pressure) in both the European and mixed ethnicity samples.

## References


[1] The COVID-19 Host Genetics Initiative. The COVID-19 Host Genetics Initiative, a global initiative to elucidate the role of host genetic factors in susceptibility and severity of the SARS-CoV-2 virus pandemic. *Eur. J. Hum. Genet.*, 28, 715–718 (2020). https://doi.org/10.1038/s41431-020-0636-6.

[2] 23andMe: DNA Genetic Testing & Analysis (2021). URL: https://www.23andme.com

[3] Barry, C., Liu, J., Richmond, R., Rutter, M. K., Lawlor, D. A., Dudbridge, F., & Bowden, J. (2020). Exploiting collider bias to apply two-sample summary data Mendelian randomization methods to one-sample individual level data. *medRxiv*.
